# Autologous periodontal stem cell assistance in periodontal regeneration technique (SAI-PRT) in the treatment of periodontal intrabony defects: A case report with one-year follow-up

**DOI:** 10.15171/joddd.2017.022

**Published:** 2017-06-21

**Authors:** Vandana KL, Haneet Ryana, Priyanka Jairaj Dalvi

**Affiliations:** ^1^Department of Periodontics, College of Dental Sciences, Davangere, Karnataka, India

**Keywords:** Osseous defects, periodontal regeneration, periodontitis, stem cells

## Abstract

Numerous animal and human studies have provided evidence supporting the belief that periodontal ligament stem cells (PDLSCs) can be harnessed for regeneration of periodontal tissues. Based on current literature on the use of ex vivo stem culture and associated problems, this case report describes a novel approach of direct application PDLSCs using stem cell assistance in periodontal regeneration technique (SAI-PRT) for the regeneration of intrabony periodontal defects bypassing ex vivo cultures. SAI-PRT has emerged as a constructive avenue in the treatment of periodontal osseous defects. Moreover, the current technique is less technique-sensitive, cost-effective and yields promising results.

## Introduction

Periodontal regeneration is challenging. The classic regeneration techniques have less predictable results. Human clinical trials using cell-based approaches for regeneration of periodontal tissues are up and coming.^1^ These host-derived stem cells can either be subjected to isolation, ex vivo expansion (stem cell culture) or re-implantation into periodontal wound/defect, or they can be injected directly as a suspension or delivered using biocompatible scaffolds or cell carriers..^2^Based on the current literature on the use of ex vivo culture and associated problems, a humble attempt was made to harvest autologous PDLSCs for direct application using Abgel®©™ (gelatin sponge-Shri Gopal Krishna Labs Pvt. Ltd. Mumbai, Maharashtra India) as scaffold in regeneration of intrabony periodontal defects bypassing ex vivo culture. We used soft tissue harboring of the PDLSCs adherent to the root of an extracted impacted wisdom tooth to restore the periodontal defect of another molar of the same patient. Stem cell assistance in the treatment of periodontal intrabony defects was attempted for the first time in the periodontal literature.

## Case report

An apparently healthy 27-year-old male patient reported to the Department of Periodontics, College of Dental Sciences, Davngere, Karnataka, with a chief complaint of food lodgment in the lower right back tooth region since two years. Periodontal findings revealed periodontal pocket distal to the mandibular right first molar and the tooth was vital. Intraoral periapical radiograph (IOPA) showed a vertical bony defect distal to mandibular right first molar up to the apical third, with periodontal space widening in the furcation. Based on this history, clinical findings and radiographic evaluation, a diagnosis of localized periodontitis was reached.

**Figure 1:**
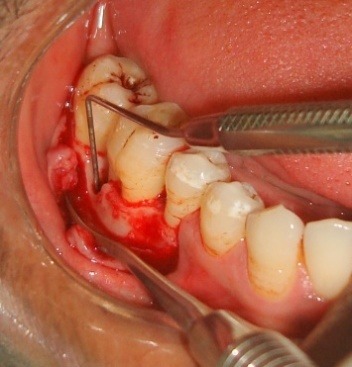
Intrabony defect distal to the right mandibular first molar.

The treatment procedure was explained to the patient and a written consent was obtained. Initial periodontal therapy included patient education, oral hygiene instructions, thorough scaling and limited occlusal adjustments. The study protocol was approved by institutional review board and was in compliance with the Helsinki Declaration. Following all the surgical protocols, a mucoperiosteal flap was raised distal to the right mandibular second premolar to the right mandibular second molar teeth two millimeter beyond the defect (Figure 1). Complete debridement of the defect was carried out followed by extraction of the impacted maxillary right third molar.

**Figure 2:**
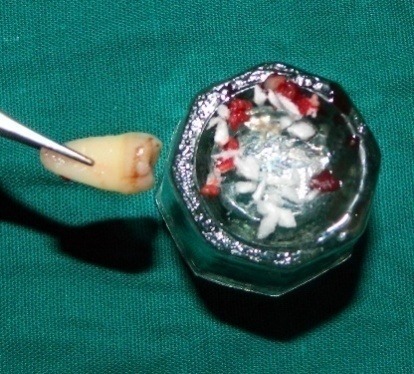
Soft tissue adherent to the root of an ex-tracted third molar, and the extraction socket harbor-ing the PDLSCs and Abgel®©™ (1×1 mm).

**Figure 3:**
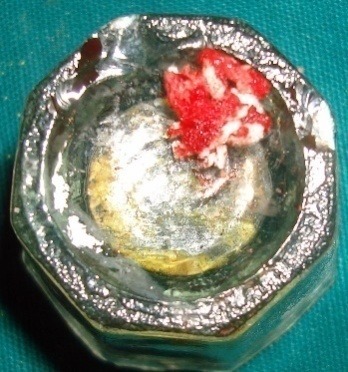
Transferable mass consisting of soft tissue adherent to the extracted third molar, cementum scraping, and scraping from the alveolar socket mixed with Abgel®©™.

The transplant consisted of soft tissue adherent to the root of an extracted third molar^3^ and the extraction socket.^4^ This tissue harbors the PDLSCs.^3,4^ Cementum scrapings were obtained by gently scraping the tooth root and the extracted socket using a sterile curette (Figure 2). Abgel®©™ was cut into small pieces (1×1 mm) and mixed with the autologous transplant tissue and cementum scrapings) in a sterile Dappen dish to obtain a transferable mass (Figure 3) to the selected intrabony defect. The soft tissue scrapings were mixed quickly with Abgel®©™ to preserve the viability of stem cells in it. The pre-sutured knot was tightened and periodontal dressing was placed. Postoperative instructions were given and the sutures were removed after 10 days.

Clinical examination was performed at baseline and at one week, three months, six months and one year postoperative intervals (Table 1). One-year follow-up revealed 6 mm of gain in the attachment level measured from a fixed reference point (stent) with a negligible change in the gingival marginal position (Table 1).

**Table 1:** Clinical measurements of the treated site distal to the mandibular right first molar at different time intervals

**Variables**	**Baseline**	**Six months**	**One year**
**PPD (Measured using UNC-15 probe)**	9 mm	4 mm	3 mm
**Gingival marginal position (GMP)**	5 mm	5 mm (4.5mm)	5 mm (4.5mm)
**Clinical attachment loss (CAL)**	8 mm	4 mm	2 mm
**Relative attachment loss using a stent as a fixed reference point (stent–FRP) and gutta-percha (GP) point (no. 80)**	16 mm	12 mm	10 mm

PPD = probing pocket depth; FRP= fixed reference point

**Figure 4:**
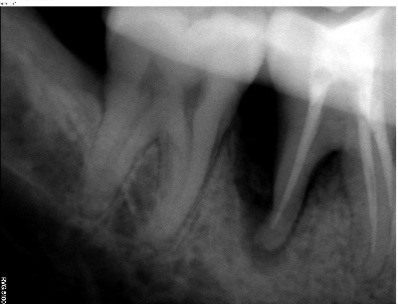
Baseline radiovisiograph (RVG) of the right mandibular first molar region; vertical bony defect distal to the mandibular right first molar up to the apical third and periodontal space widening in the furcation area.

**Figure 5:**
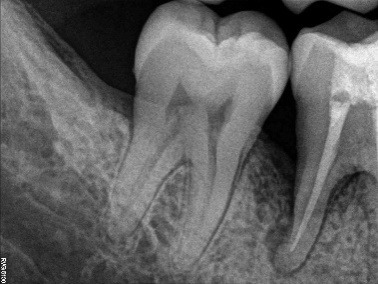
Radiovisiograph (RVG) of the right mandibular first molar region at one-year follow-up; radiographic defect fill seen distal to the right mandibular first molar.

Radiographic evaluations were performed at baseline, six months and one year. The radiographic images were measured using CorelDraw Graphics Suite X6 and the density changes were measured using Adobe Photoshop CS3. The percentage of the defect fill was 44.5% and there was a change in radiodensity of the defect area, suggestive of improvement in newly formed bone (Figures 4 and 5).

## Discussion

PDLSCs are multipotent cells and under the influence of proper growth factors can differentiate into osteoblasts, fibroblasts and cementoblasts to form PDL-like tissues.^3^ Cemental matrix is a rich source of many growth factors such as IGF, FGF, BMPs and many more, which influence the activities of various periodontal cell types.^5^ A scaffold or a carrier material helps in the delivery of stem cells and various angiogenic factors to the site where periodontal regeneration is desired.^6^ In the current case a gelatin sponge which was biocompatible and biodegradable and also supported osteoblasts, known to promote bone regeneration in osseous defects, was considered.^7^In the first human clinical trial, autologous PDL-derived cells, including PDLSCs, were cultured (ex vivo) and then transplanted^1^ for the treatment of periodontal osseous defects in three patients.^1^ However, ex vivo cell culture techniques are costly and sensitive and there is a chance of loss of stem cells during cell passage, genetic alteration and tumorogenic potential.^8^

Based on the current literature on the use of ex vivo culture and associated problems, a humble attempt was made to harvest autologous PDLSCs for direct application using Abgel®©™^**^ (gelatin sponge) as a scaffold in regeneration of intrabony periodontal defects, bypassing ex vivo culture. This technique was tried for the first time in the periodontal literature and it abides by tissue engineering triad^9^ of PDLSCs (cells), Abgel®©™^**^ (gelatin sponge scaffold) and cementum scrapings (signaling molecules).^5^ Stem cell assistance in periodontal regeneration technique (SAI-PRT) has resulted in successful clinical and radiographic parameters such as clinical attachment gain, decreased probing pocket depth and satisfactory defect fill of intrabony defects when evaluated  for a period of one year. The immediate periodic healing events were uneventful. It can be proposed that periodontal stem cells from tissue adherent to third molar may have contributed to regeneration. The radiographic images were measured using Corel Draw X6 and the density changes were measured using Adobe Photoshop CS3 for the first time in the literature. The influence of gingival margin position (GMP) on pocket depth improvement was minimal due to negligible changes in GMP as the apical shift in the gingiva exaggerates PPD reduction postoperatively.^10^

## Conclusion

Although direct application of PDL stem cell therapy looks promising, it is still in its stage of infancy, and more work is needed in this area to validate the results. Stem cell assistance in periodontal regeneration technique (SAI-PRT) could overcome the limitations and concerns of ex vivo cell culture techniques as they are costly and sensitive and there is chance of loss of stem cells during cell passage, genetic alteration and tumorogenic potential.^8^A simple task of PDLSCs procurement and immediate placement is the major advantage of the current concept. The autologous stem cell assistance in periodontal regeneration technique (SAI-PRT) has emerged as a constructive avenue in the treatment of periodontal osseous defects. At present the limitations of the study are the uncertainty on the number and viability of cells transplanted immediately after scraping the tissues from the root surfaces of extracted teeth. As of now lack of histological evidence and in vitro analysis of stem cell characterization and osteogenic potential are some important shortcomings. Further studies are being directed wherein 15 patients are undergoing current therapy as randomized controlled trail with due consideration of the above shortcomings. SAI-PRT is clinically feasible and cost-effective compared to currently available techniques; therefore, the clinical application of this novel idea is recommended.
